# Diagnostic accuracy of deep-learning with anomaly detection for a small amount of imbalanced data: discriminating malignant parotid tumors in MRI

**DOI:** 10.1038/s41598-020-76389-4

**Published:** 2020-11-09

**Authors:** Hidetoshi Matsuo, Mizuho Nishio, Tomonori Kanda, Yasuyuki Kojita, Atsushi K. Kono, Masatoshi Hori, Masanori Teshima, Naoki Otsuki, Ken-ichi Nibu, Takamichi Murakami

**Affiliations:** 1 Department of Radiology, Kobe University Graduate School of Medicine, Kobe, Japan; 2 Department of Otolaryngology-Head and Neck Surgery, Kobe University Graduate School of Medicine, Kobe, Japan

**Keywords:** Cancer, Software

## Abstract

We hypothesized that, in discrimination between benign and malignant parotid gland tumors, high diagnostic accuracy could be obtained with a small amount of imbalanced data when anomaly detection (AD) was combined with deep leaning (DL) model and the L_2_-constrained softmax loss. The purpose of this study was to evaluate whether the proposed method was more accurate than other commonly used DL or AD methods. Magnetic resonance (MR) images of 245 parotid tumors (22.5% malignant) were retrospectively collected. We evaluated the diagnostic accuracy of the proposed method (VGG16-based DL and AD) and that of classification models using conventional DL and AD methods. A radiologist also evaluated the MR images. ROC and precision-recall (PR) analyses were performed, and the area under the curve (AUC) was calculated. In terms of diagnostic performance, the VGG16-based model with the L_2_-constrained softmax loss and AD (local outlier factor) outperformed conventional DL and AD methods and a radiologist (ROC-AUC = 0.86 and PR-ROC = 0.77). The proposed method could discriminate between benign and malignant parotid tumors in MR images even when only a small amount of data with imbalanced distribution is available.

## Introduction

Most parotid tumors are benign, but a small number of malignancies can occur^1^. Malignant parotid tumors have diverse histological characteristics, and therefore an accurate diagnosis by imaging alone is difficult. In addition, the accuracy of benign/malignant discrimination using fine-needle aspiration cytology is low^2^ and involves the risk of tumor seeding^3^. Thus, further improvement of imaging diagnosis is desired.

In recent years, it has been reported that the diagnostic ability of artificial intelligence (AI) systems, such as deep neural networks, that is, deep learning (DL), is comparable to or exceeds that of specialists in several medical fields^4,5^. Even though DL models constructed using a large amount of data have achieved promising results, it is difficult to obtain an accurate AI model through DL using small or imbalanced datasets. Generally, medical image data are difficult to handle owing to the protection of personal information. Furthermore, collecting a sufficient number of cases requires a considerable amount of time, resulting in small datasets. Therefore, it is difficult to construct an accurate DL model for clinical images of parotid tumors.

We hypothesized that this issue could be resolved by combining feature extraction through DL with anomaly detection (AD), which is often used for imbalanced datasets. In addition, to ensure robustness, non-medical images were used to train a DL model. The purpose of this study was to differentiate magnetic resonance (MR) images of benign/malignant parotid tumors using DL with AD.

Our contributions of this study are summarized as follows. We examined a combination of DL and AD for a relatively small dataset with a highly imbalanced distribution. As an example of a small dataset with imbalanced distribution, we used a relatively small dataset of MR images to discriminate between benign and malignant parotid tumors. To construct a robust and reliable DL model using the dataset, the L_2_-constrained softmax loss was used for the optimization target. In addition, non-medical images were used for data augmentation. These techniques prevented the model from overfitting in the small dataset. In this method, graphics processing unit (GPU) acceleration was not used, and the training of the model was completed in a reasonable amount of time.

## Theoretical framework

Diagnostic accuracy equivalent to that of dermatologists was achieved by using a convolutional neural network for the classification of skin cancer^5^. The application of DL to histopathological tissue samples has been advanced, and DL was used for the discrimination of malignant lymphoma^6^ and breast cancer^7^. Although the detection of malignant thyroid and salivary gland tumors using DL has already been reported for histopathological samples^8^, there have been no reports on DL for the discrimination of parotid gland tumors using MRI images, except for reports on texture analysis^9,10^.

Training a conventional machine learning algorithm using a small amount of data has achieved promising results^4^. However, there are only a few reports on DL^11^. It is generally known that a large amount of training data can lead to better performance, whereas training a DL model on a small amount of data may be difficult. Johnson et al. investigated the use of DL in a large amount of highly imbalanced data^12^. However, the application of DL to a small amount of highly imbalanced data is still unknown.

## Materials and methods

This study was conformity with the Declaration of Helsinki and Ethical Guidelines for Medical and Health Research Involving Human Subjects in Japan (https://www.mhlw.go.jp/file/06-Seisakujouhou-10600000-Daijinkanboukouseikagakuka/0000080278.pdf). The requirement to obtain informed consent was waived because of the retrospective design. This study was approved by the Kobe University Ethical Committee (Permission number: B190167) and carried out according to the guidelines of the committee.

### Sample

Magnetic resonance images (T1- and T2-weighted images) of 245 parotid tumors obtained between April 2010 and March 2019 were retrospectively collected in a single center. Patient age ranged from 11 to 86 years (with a median of 56 years). A total of 122 cases were male, and 123 were female. Tumor histopathology was confirmed by surgery, whereby 190 (77.6%) tumors were classified as benign, and 55 (22.4%) as malignant (Table 1).

**Table 1:** Subtypes of parotid tumors and data distribution of the train, validation, and test sets.

Pathological type	Training	Validation	Test	Total
**Benign tumor**	116	38	36	190
Pleomorphic adenoma	81	25	26	132
Warthin’s tumor	18	6	5	29
Other benign tumors	17	7	5	29
**Malignant tumor**	35	10	10	55
Mucoepidermoid carcinoma	13	3	3	19
Acinic cell carcinoma	5	2	1	8
Malignant lymphoma	5	1	1	7
Other malignant tumors	12	4	5	21
Total cases	151	48	46	245

**Figure 1:**
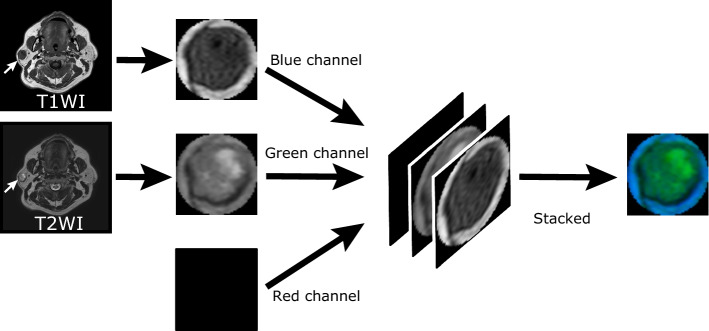
Generation of pseudo-color image from MR images. For each case, T1- and T2-weighted grayscale images with the largest axial cross-section of the tumor are cropped. To obtain input to a DL model from the MR images, the cropped T1- and T2-weighted grayscale images are fed to the blue and green channels, respectively, of a pseudo-color image (RGB image). The red channel of the pseudo-color image is empty.

**Figure 2:**
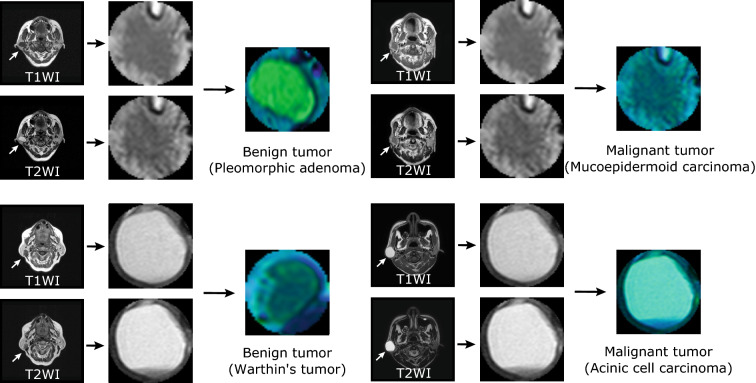
Examples of MR images of benign and malignant tumors. Original MR images (T1- and T2-weighted images) and their pseudo-color images of benign and malignant tumors.

The main parameters of MR imaging were as follows: The magnetic field strength was primarily 1.5 and 3 T (data distribution is shown in Table 2), slice thickness was 1.5–10 mm, and the matrix size was ranged from 256 × 256 to 960 × 960. For each case, T1- and T2-weighted grayscale images were cropped by a board-certified radiologist (17 years of experience), resulting in images with the largest axial cross-section of the tumor. To obtain input to the DL model from the MR images, the cropped T1- and T2-weighted grayscale images were fed to the blue and green channels of a pseudo-color image (RGB image), respectively. The red channel was empty. Thereby, 245 pseudo-color images were obtained from the MR images, and they were scaled to fit the input size of the DL model (Figs. 1 and 2).

**Table 2:** Magnetic field strength of the MRI equipment.

Magnetic field strength (T)	Cases
1.5	204
3.0	35
Others	6
Total cases	245

The dataset consisting of these pseudo-color images was randomly divided into the training (60%), validation (20%), and test set (20%). In the three sets, benign and malignant images were distributed in equal proportions.

### Data analysis procedures

**Figure 3:**
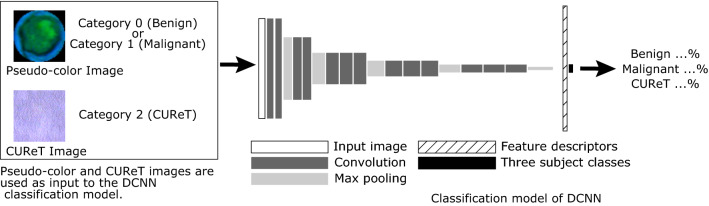
Scheme of the proposed DCNN classification model (VGG16). Regarding the pseudo-color MR image, the categories corresponding to benign and malignant tumors are 0 and 1, respectively. The category corresponding to CUReT images is 2. Training is performed as three-class classification using the DCNN classification model based on VGG16; its output is the probability of category 0 (benign), category 1 (malignant), or category 2 (CUReT). *DCNN* deep convolutional neural network, *CUReT* Columbia–Utrecht reflectance and texture database.

In this study, a two-stage model was used: The first stage involved a classification model using a deep convolutional neural network (DCNN), whereas in the second stage, an outlier detection method was employed for AD. In the former, a DCNN based on VGG16^13^ was used for classification, and transfer learning was performed^14^. Then, the output of the DCNN before the final output layer (feature descriptors) was fed to the local outlier factor (LOF), which was used for outlier detection^15^. The LOF classified the DCNN feature descriptor as normal (corresponding to benign) or abnormal (corresponding to malignant) (Fig. 3).

We used VGG16 attached to Keras (https://github.com/fchollet/keras) as a DCNN classification model, as suggested by the Visual Geometry Group at the University of Oxford in ILSVRC2014. We changed the input image size of VGG16 to 100 × 100 pixels, and the loss function was the L_2_-constrained softmax, as described in the below. The L_2_-constrained softmax loss forces the length of feature descriptors ${\varvec{x}}$ to be a pre-specified constant ($\alpha$):

$${\text{feature descriptors }}\user2{x}{\text{ }} \to {\text{ }}\frac{{\alpha \user2{x}}}{{\left\| \user2{x} \right\|}}.$$

All processing was performed on a PC without a discrete GPU (Core i5 5257U CPU at 2.7 GHz, RAM 8 GB). Python (version 3.6.8) (https://www.python.org) was used as the programing language, and Keras (version 2.1.6) and TensorFlow (version 1.13.2) (https://tensorflow.org/) were used as the deep learning framework. Adam, with a learning rate of 1.0 × 10^–5^, was used as the optimizer^16^. Transfer learning was performed by freezing the trainable parameters of 10 layers in VGG16, including the convolutional layer. The network was trained using a batch size of 1 and up to 500 epochs. The training stopped when the loss of the validation set was not improved. In each epoch, all the pseudo-color images of training set as well as the non-medical images from the CUReT dataset were processed. The details of the CUReT dataset are described in the following paragraph. The execution of all training processes on the PC required approximately half a day.

It is well known that the softmax loss is effective on high-quality datasets with small data variation. However, when the data are imbalanced and of poor quality, performance degradation occurs. On the other hand, the L_2_-constrained softmax loss is known to be effective even for low-quality data with strong imbalance^17^. Specifically, on a hypersphere, minimizing the L_2_-constrained softmax loss is equivalent to maximizing the cosine similarity for the same category pairs, and minimizing it for different category pairs, thus strengthening the feature verification signal. Moreover, the L_2_-constrained softmax loss can better classify extreme and difficult images because all the feature descriptors have the same L_2_-norm^17^. The L_2_- constrained softmax loss is given by

1 $$\begin{gathered} {\text{minimize}} - \frac{1}{{\varvec{M}}}\mathop \sum \limits_{{{\varvec{i}} = 1}}^{{\varvec{M}}} {\text{log}}\frac{{{\varvec{e}}^{{{\varvec{W}}_{{{\varvec{y}}_{{\varvec{i}}} }}^{{\varvec{T}}} {\varvec{f}}\left( {{\varvec{x}}_{{\varvec{i}}} } \right) + {\varvec{b}}_{{{\varvec{y}}_{{\varvec{i}}} }} }} }}{{\mathop \sum \nolimits_{{{\varvec{j}} = 1}}^{{\varvec{C}}} {\varvec{e}}^{{{\varvec{W}}_{{\varvec{j}}}^{{\varvec{T}}} {\varvec{f}}\left( {{\varvec{x}}_{{\varvec{i}}} } \right) + {\varvec{b}}_{{\varvec{j}}} }} }} \hfill \\ \begin{array}{*{20}c} {{\text{subject to}} \parallel f\left( {{\varvec{x}}_{{\varvec{i}}} } \right)\parallel_{2} = \alpha ,\user2{ }\forall_{{\varvec{i}}} = 1,2 \ldots M } \\ \end{array} \hfill \\ \end{gathered}$$

where $x_{i}$ is the input image in a batch of size $M$, $y_{i}$ is the corresponding class label, and $f\left( {x_{i} } \right)$ is the feature descriptor obtained from the penultimate layer of the DCNN classification model. $C$ is the number of classes, and $W$ and $b$ are the weights and bias, respectively, for the last layer of the network, which acts as a classifier. In the proposed model, $M = 1$, $C = 3$, and $\alpha$ was set to 80.

**Figure 4:**
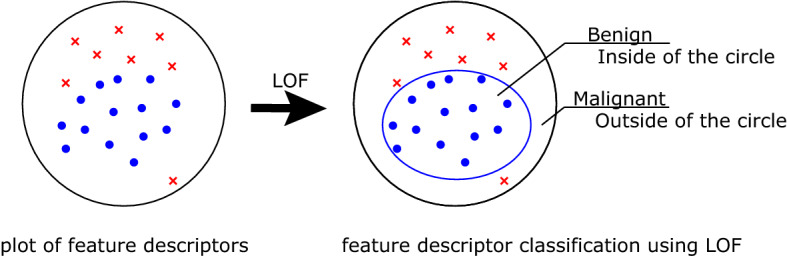
Schematic illustration of L_2_-constrained softmax and LOF. For each case, a feature descriptor is obtained from the DCNN. The left part shows a plot of the feature descriptors. LOF can be used to determine the threshold of benign tumors (inside the circle) based on the local density of the training dataset, and discriminate whether the test dataset are normal (benign) or abnormal (malignant). *LOF* local outlier factor.

In this study, the output of the classification model of a DCNN with the L_2_-constrained softmax loss before the final output layer (feature descriptor) was fed to the LOF (an abnormality detection method) to classify MR images into two groups: normal (benign) and abnormal (malignant). The LOF is an unsupervised AD method that computes the local density deviation of a given data point with respect to its neighbors^15^. Implementation of Scikit-learn was used for the LOF in this study^18^. The brute-force search method was used to compute the nearest neighbors, and the squared Euclidean distance was used for the distance computation. The local density is required to calculate the LOF. The details of the local density are as follows^15^:

2 $$\begin{array}{*{20}c} {{\text{lrd}}\left( {\varvec{p}} \right) = {\raise0.7ex\hbox{$1$} \!\mathord{\left/ {\vphantom {1 {\frac{{\mathop \sum \nolimits_{{{\varvec{q}} \in {\varvec{N}}_{{\varvec{k}}} \left( {\varvec{p}} \right)}} {\varvec{d}}\left( {{\varvec{p}},{\varvec{q}}} \right)}}{{\left| {{\varvec{N}}_{{\varvec{k}}} \left( {\varvec{p}} \right)} \right|}}}}}\right.\kern-\nulldelimiterspace} \!\lower0.7ex\hbox{${\frac{{\mathop \sum \nolimits_{{{\varvec{q}} \in {\varvec{N}}_{{\varvec{k}}} \left( {\varvec{p}} \right)}} {\varvec{d}}\left( {{\varvec{p}},{\varvec{q}}} \right)}}{{\left| {{\varvec{N}}_{{\varvec{k}}} \left( {\varvec{p}} \right)} \right|}}}$}}} \\ \end{array}$$

where ${\text{lrd}}\left( p \right)$ is the local density of object $p$ ($p$) is represented by the feature descriptor obtained from the DCNN), $N_{k} \left( p \right)$ is the $k$-distance neighborhood of $p$, and $d\left( {p,q} \right)$ is the distance between objects $p$ and $q$ in the specified space (in this study, the squared Euclidean distance). Using ${\text{lrd}}\left( p \right)$, the LOF of $p$ is defined as follows:

3 $$\begin{array}{*{20}c} {{\text{LOF}}\left( {\varvec{p}} \right) = \frac{{\mathop \sum \nolimits_{{{\varvec{q}} \in {\varvec{N}}_{{\varvec{k}}} \left( {\varvec{p}} \right)}} \frac{{{\text{lrd}}\left( {\varvec{q}} \right)}}{{{\text{lrd}}\left( {\varvec{p}} \right)}}}}{{\left| {{\varvec{N}}_{{\varvec{k}}} \left( {\varvec{p}} \right)} \right|}} ,} \\ \end{array}$$

where $k$ was set to 5 in this study. The output LOF value was used to determine whether $p$ (tumor) was normal (benign) or abnormal (malignant) (Fig. 4).

**Figure 5:**
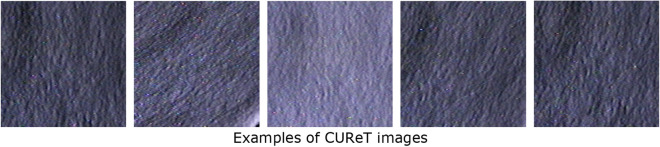
Example images from CUReT dataset. For DCNN training, the center of the CUReT image is cropped to fit the input size of the DCNN.

To improve the robustness of feature extraction in the DCNN classification model, images from the Columbia–Utrecht reflectance and texture database (CUReT)^19^, which is often used as an artificial texture library, were used as a third class in addition to benign and malignant tumors (Figs. 3 and 5). This technique can be regarded as data augmentation by adding a non-medical image dataset to a medical image dataset.

A combination of conventional augmentation (vertical and horizontal flip, rotation by − 180° to + 180°), random erasing^20^, and mix-up^21^ were also performed in every training epoch in the proposed method.

In addition to the proposed method, we evaluated the performance of several DL models and other AD methods, such as the DCNN classification model alone, a combination of the DCNN classification model and the other AD method (one-class support vector machine (OCSVM)), and convolutional variational autoencoder (CVAE)^22^. CVAE is a type of autoencoder used for AD. In addition to VGG16^13^, we used MobileNet^23^ and ResNet50^24^ as DCNN classification models. They were included in Keras, and their input image sizes were modified in the current study. In addition to the L_2_-constrained softmax loss, the conventional softmax loss was also used in the DL models. After the output of the DCNN classification model was obtained as a feature descriptor, OCSVM^25^, which is commonly used for AD, was also evaluated instead of the LOF. As shown, in addition to benign and malignant images, images from the CUReT dataset were used as a third class in the proposed method. For comparison, the results of training DL models without the CUReT dataset were also evaluated.

### Measures

Using the test dataset, we evaluated the diagnostic accuracy of the proposed DL with AD, conventional two-categorical DCNN classification models, and CVAE. In addition to the radiologist who cropped the images, another board-certified radiologist with 18 years of experience evaluated his diagnostic accuracy by using a 5-point scale: 1 (definitely benign) to 5 (definitely malignant). Receiver operating characteristic curve (ROC) analysis and precision-recall (PR) analysis were performed, and the area under the curve (AUC) was calculated for the results by the models and by the radiologist. The sensitivity and specificity values were calculated using the threshold obtained by the Youden index. The 95% confidence interval (95%CI) of sensitivity, specificity, and PR-AUC was calculated by the bootstrap method. The 95%CI of ROC-AUC was calculated by the Delong method.

## Results

**Figure 6:**
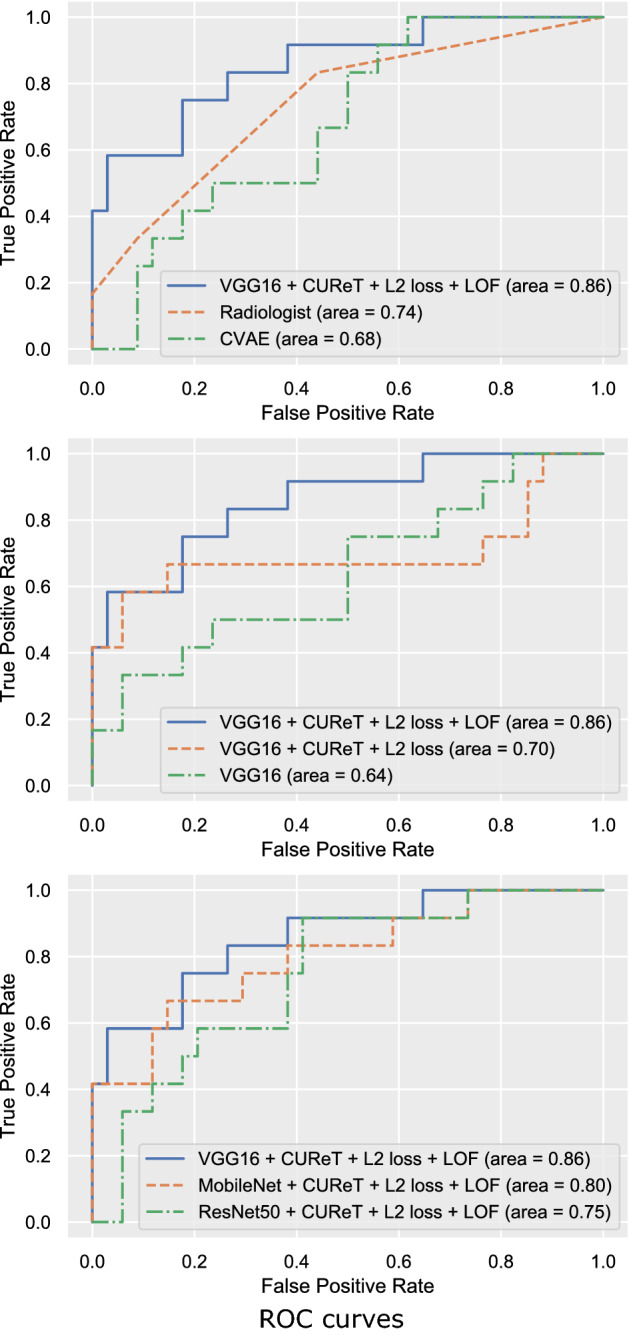
ROC Curves (N = 46). The top shows a comparison between the proposed model, a radiologist, and CVAE. The middle shows the comparison between the three methods using VGG16 as a DCNN. The bottom shows a comparison between the three DCNN models using LOF for AD. *L2 loss* L_2_-constrained softmax loss, *CVAE* convolutional variational autoencoder.

The diagnostic accuracy of the models evaluated in this study is shown in Table 3. The VGG16-based model with the L_2_-constrained softmax loss and LOF exhibited the highest diagnostic performance (ROC-AUC = 0.86 and PR-ROC = 0.77) (Fig. 6). The combined use of the CUReT dataset, L_2_-constrained softmax loss, and LOF resulted in the highest diagnostic performance among all DCNN classification models. In VGG16, the addition of the CUReT dataset resulted in an evident improvement of the diagnostic performance, whereas changing the loss function to the L_2_-constrained softmax loss resulted in a reduction. However, the combined use of the CUReT dataset with AD further improved the performance (Table 3). For ResNet50 and MobileNet, a slight improvement by the addition of the CUReT dataset was observed. Table 3 also shows that in each of the three models, the combination of the LOF and AD yielded better results than the combination of OCSVM. The combination of the LOF and CUReT yielded better results than the addition of the CUReT dataset alone. The VGG16 network tended to yield better results than MobileNet and ResNet50 (Fig. 6). The radiologist performed better than CVAE, but worse than the VGG16-based model with the L_2_-constrained softmax loss and LOF (Fig. 6).

**Table 3:** Classification results of different networks (N = 46).

Network model	Sensitivity	Specificity	ROC-AUC	PR-AUC
**Classification model of DCNN**				
**VGG16**				
Conventional	0.42 (0.17–0.75)	0.82 (0.68–0.94)	0.64 (0.45–0.84)	0.50 (0.25–0.78)
+ CUReT	0.67 (0.42–0.92)	0.94 (0.91–0.97)	0.81 (0.65–0.98)	0.75 (0.52–0.97)
+ CUReT + L_2_-constrained softmax loss	0.58 (0.25–0.83)	0.94 (0.85–1.00)	0.70 (0.47–0.93)	0.68 (0.45–0.92)
+ CUReT + L_2_-constrained softmax loss + OCSVM	0.58 (0.33–0.83)	0.94 (0.85–1.00)	0.77 (0.57–0.97)	0.71 (0.50–0.94)
+ CUReT + L_2_-constrained softmax loss + LOF	0.75 (0.50–1.00)	0.82 (0.68–0.94)	**0.86** (0.73–0.99)	**0.77** (0.57–0.98)
**MobileNet**				
Conventional	0.50 (0.25–0.83)	0.91 (0.82–1.00)	0.60 (0.38–0.83)	0.50 (0.24–0.77)
+ CUReT	0.67 (0.42–0.92)	0.65 (0.47–0.79)	0.70 (0.52–0.88)	0.52 (0.30–0.76)
+ CUReT + L_2_-constrained softmax loss	0.41 (0.17–0.67)	0.85 (0.73–0.97)	0.57 (0.34–0.79)	0.48 (0.24–0.72)
+ CUReT + L_2_-constrained softmax loss + OCSVM	0.66 (0.42–0.92)	0.88 (0.76–0.97)	0.79 (0.63–0.95)	0.63 (0.40–0.89)
+ CUReT + L_2_-constrained softmax loss + LOF	0.66 (0.42–0.92)	0.85 (0.74–0.97)	0.80 (0.65–0.96)	0.70 (0.49–0.93)
**ResNet50**				
Conventional	0.67 (0.53–0.82)	0.68 (0.53–0.82)	0.51 (0.29–0.73)	0.38 (0.14–0.56)
+ CUReT	0.56 (0.38–0.71)	0.83 (0.58–1.00)	0.74 (0.58–0.90)	0.47 (0.24–0.77)
+ CUReT + L_2_-constrained softmax loss	0.58 (0.33–0.83)	0.68 (0.50–0.82)	0.51 (0.29–0.73)	0.31 (0.14–0.56)
+ CUReT + L_2_-constrained softmax loss + OCSVM	0.91 (0.75–1.00)	0.50 (0.32–0.68)	0.70 (0.54–0.86)	0.42 (0.22–0.72)
+ CUReT + L_2_-constrained softmax loss + LOF	0.92 (0.75–1.00)	0.59 (0.44–0.73)	0.75 (0.59–0.90)	0.47 (0.26–0.78)
**CVAE**	1.00 (1.00–1.00)	0.38 (0.24–0.56)	0.68 (0.52–0.84)	0.39 (0.20–0.67)
**Radiologist**^**a**^	0.83 (0.58–1.00)	0.56 (0.38–0.71)	0.74 (0.58–0.90)	0.51 (0.29–0.76)

*ROC-AUC* area under the curve of receiver operating characteristic curves, *PR-AUC* area under the curve of precision-recall curves, *DCNN* deep convolutional neural network, *CUReT* Columbia-Utrecht Reflectance and Texture Database, *OCSVM* one class support vector machine, *LOF* local outlier factor, *CVAE* convolutional variational autoencoder.

^a^The radiologist was board-certified.

## Discussion

Two-category classification has often been used for classifying objects into two groups (e.g., benign and malignant). However, training DCNN classification models on two categories using a small dataset is known to lead to overfitting. In the present study, even though only a small amount of training data could be used, a significant performance improvement was obtained by including images from the CUReT dataset as a third category, in addition to the data augmentation. This is because the addition of the CUReT dataset facilitated the extraction of general visual patterns and reduced the overfitting caused by the small number of images.

In this study, we used the L_2_-constrained softmax loss, which was demonstrated to be superior to the categorical softmax loss in extracting features from small and imbalanced datasets. By training the DCNN classification model using this loss function, the feature descriptors of less diverse and more numerous benign tumors were dense in the feature space, and those of more diverse and less numerous malignant tumors were sparse and far from benign tumors. These characteristics of the feature descriptors suggest that the use of the LOF and AD based on local density and distance is quite effective. Accordingly, it may be possible to improve the accuracy of DL by combining AD techniques (LOF) with DCNNs.

In this study, we did not use GPUs, which are commonly employed in training DL models; rather, we used CPUs, which are commonly employed in general applications, for DL training and evaluation. Nevertheless, the results demonstrated that the diagnostic accuracy of the proposed model was at least comparable to that of the radiologist. The computational performance was improved owing to the smaller resolution of the input images resulting from cropping only tumor images, the use of a relatively small DCNN, and the smaller number of input images. Among the DCNN classification models, VGG16 generally performed better than MobileNet and ResNet50. This was inconsistent with the number of modifiable parameters (VGG16, 138,357,544; MobileNet, 4,253,864; and ResNet50, 25,636,712)^27^, and with the ImageNet-based evaluation (Top1 accuracy VGG16, 0.713; MobileNet, 0.665; and ResNet50, 0.759)^27^. On the other hand, the performance excellence of VGG-16 is attributed to the fact that the architecture depth of VGG-16 is optimal to learn from the dataset of the current study. Optimal DCNNs for medical images characterized by low-resolution and imbalanced datasets should be investigated in future work.

The accuracy of benign/malignant discrimination using ultrasound is reportedly low, even with pulsed Doppler and color Doppler sonography (sensitivity 72%; specificity 88%)^28^. It has been reported that no clear correlation between malignancy and the characteristics of MR images has been found in non-contrast MR imaging. Moreover, the use of dynamic MR images and the apparent diffusion coefficient improved the accuracy of benign/malignant discrimination (sensitivity, 86%; specificity, 92%)^29^. Our results indicate lower diagnostic values, with a sensitivity of 75% and a specificity of 82%. Diagnostic accuracy is affected by the pretest probability of the disease in the studies; thus, it is not easy to compare only the values themselves among different studies. To resolve this issue, in the present study, the diagnostic accuracy of the radiologist was evaluated using image-interpretation experiments, and it was demonstrated to be lower than that of the proposed DL model.

Generally, the construction of DL models with high diagnostic performance requires a large amount of image data. The use of a small or imbalanced dataset may be considered a challenging task for constructing robust and reliable DL models. The results of this study demonstrated that the proposed technique enabled the construction of a DL model in the case of a small number of images. In the medical field, as it is often difficult to collect big and balanced data for rare diseases, the proposed technique may facilitate the construction of a robust DL model with high diagnostic performance. In addition, even though we only used a CPU without a GPU, we obtained satisfactory results. The proposed method has a relatively low computational cost and may therefore be easier to implement than complex and large DCNN models.

## Limitations and conclusion

Our study has several limitations. Specifically, the pseudo-color images were created by selecting only the slice containing the maximum diameter of the tumor, and this slice may not best represent the characteristics of the tumor. The most important aspect in the actual MRI diagnosis is the invasiveness of the surrounding area observed in malignant tumors^1^. In this study, however, such invasiveness was not necessarily included in the evaluation slice, and thus the diagnostic accuracy might differ from that of a clinical diagnosis. Furthermore, although 3D data may improve diagnostic accuracy, we only constructed a 2D artificial intelligence model, as a 3D model would require extensive image processing. Finally, the CUReT dataset, which is often used in texture analysis, was used as the non-medical image dataset in this study. It is necessary to investigate whether other non-medical images can be used to prevent overfitting in small datasets.

In conclusion, the proposed method (i.e., a combination of DL with AD) could discriminate between benign and malignant parotid tumors in MR images even though the DL training data consisted of a small number of images with strongly imbalanced distribution. Among the various DL models and AI techniques, the VGG16-based model with the L_2_-constrained softmax loss, LOF, and CUReT datasets exhibited the highest diagnostic accuracy. As a potential application of the proposed method, it may be possible to obtain an accurate and robust DL model in diseases for which it has been difficult to construct a DL model due to a small amount of data with imbalanced distribution.
